# Isolated Hepatic Tuberculoma – A Case Report

**Published:** 2016-12-24

**Authors:** Biplab Kr Biswas, Subrata Pal, Dhruba Jyoti Moulik, Mrinal Sikdar

**Affiliations:** 1 *Dept. of Pathology, Bankura Sammilani Medical College,* *West Bengal, India*; 2 *Dept. of Pathology, College of Medicine and Sagore Dutta Hospital,* *West Bengal, India*; 3 *Dept. of Surgery, Bankura Sammilani Medical College, West Bengal, India*; 4 *Dept. of Pathology, Kolkata National Medical College, West Bengal, India*

**Keywords:** Hepatic Tuberculoma, Nodular Mass Lesion, India

## Abstract

Isolated hepatic tuberculoma is localized hepatic tuberculosis (TB) without bile duct involvement, which presents as solitary or multiple nodular mass lesion of liver mimicking a neoplastic lesion in radiological evaluation. Clinical presentation and biochemical tests for liver functions show non-specific abnormality, which is not helpful for diagnosis. As the treatment, modality of isolated hepatic tuberculoma is anti-tubercular drugs. Prognosis is very good in comparison to other differential diagnoses. We are presenting such a rare case of isolated hepatic tuberculoma from tribal area of Bankura district, West Bengal, India in a 38-yr female patient presenting as fever, abdominal pain and solitary nodular lesion on radiological evaluation. Even different imaging modalities cannot make accurate diagnosis of isolated hepatic tuberculoma where simple biopsy and histopathology of the lesion can confirm the diagnosis.

## Introduction

Tuberculosis (TB) may involve liver in three types: 1) Secondary to miliary TB (commonest form accounts 50-80% cases); 2) Granulomatous disease (tuberculous hepatitis) due to TB; and 3) Localized hepatic TB with or without bile duct involvement (1, 2). Isolated hepatobiliary TB is until uncommon and accounts <1% of all TB (1, 3). Either localized hepatic TB presents without bile duct involvement causing solitary or multiple nodular mass lesion in liver parenchyma or it involves bile ducts causing obstructive jaundice (2). Isolated hepatic tuberculoma is the rarest type tuberculous lesion in liver and it often mimics a neoplastic liver disease (primary or solitary metastatic nodule) (4). 

Treatment of isolated hepatic tuberculoma is anti-tubercular drugs whereas other neoplastic mimickers need surgical or chemotherapeutic intervention. Sometimes, imaging modalities cannot differentiate the hepatic tuberculoma from other neoplastic mimickers; however, biopsy and histopathology can diagnose it correctly.

We are presenting a rare case of isolated hepatic tuberculoma presenting as a solitary nodular lesion in a 38-year female patient.

## Case report

A 38 yr old female patient from tribal area of Bankura district, West Bengal, India was admitted in surgery ward with complaints of pain at right hypochondrium, anorexia and weight loss and occasional fever for 3 wk. She had no history of exposure to tuberculosis. She had average built and vitals were stable. On physical examination, she had mild hepatomegaly and mild pallor. Routine blood examination revealed hemoglobin level 8.9% gm. and erythrocyte sedimentation rate (ESR) - 65mm/hr. Among liver function tests (LFT), patient had hypoalbuminaemia (3 gm/d1 and hyper-globulinemia (3.8 gm/d1), with hyper-bilirubinaemia (2.5 mg/d1, direct 1.7 mg/d1), elevated SGOT 94U/L and alkaline phosphatase 279U/L. Viral serological tests were negative for hepatitis and HIV virus. Blood biochemistry and renal function tests were in normal range. Informed consent was taken from the patient.

Chest X-ray of the patient had no abnormality. Ultrasound examination of the whole abdomen done and it revealed a 2.3 cm hypoechoic nodule at right lobe of liver and two small periportal lymph nodes of 0.8 cm and 0.5 cm diameter. On CT scan, liver was mild enlarged and there was a 2.3 cm X 1.8 cm hypoechoic nodule (SOL) at right lobe of liver without central necrosis. There were two small periportal lymph nodes but no perihepatic collection was found. CT guided FNAC was done from liver nodule and it revealed necrotic material only. The Ziehl–Neelsen stain from aspirated material did not reveal acid-fast bacilli. 

**Fig. 1:**
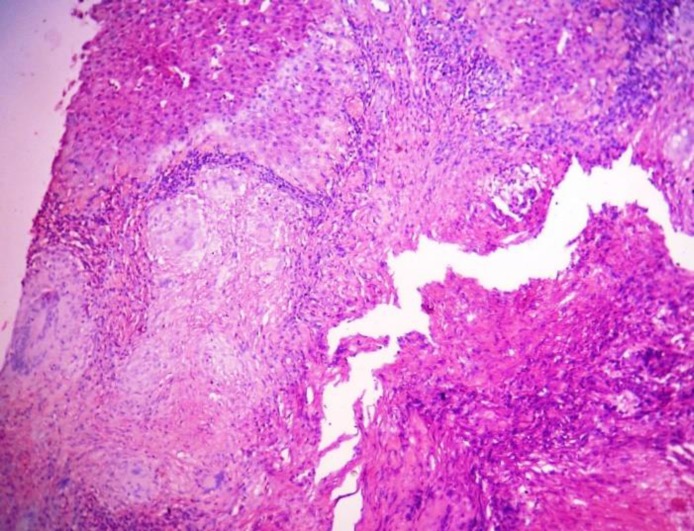
Photomicrograph shows epithelioid granulomas and Langhans-type giant cells surrounded by lymphocytes, histomorphology of TBof liver (H & E stain, 10X view

**Fig. 2:**
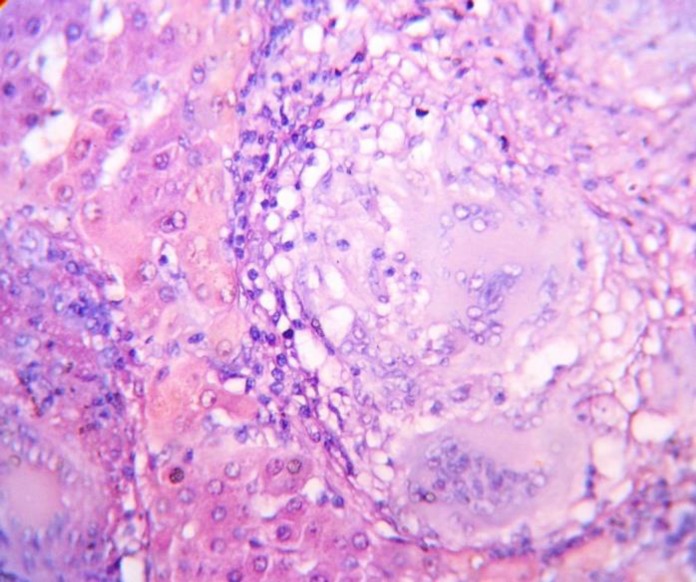
Photomicrograph shows classical epithelioid granulomas and Langhans-type giant cells in histomorphology of TBof liver (H & E stain, 40X view

The etiological diagnosis could not be clinched after routine diagnostic modalities. Diagnostic laparoscopy was done and biopsy was taken from the liver nodule. Histopathological examination of the biopsy specimen showed multiple well-defined epithelioid granulomas, caseation necrosis, and many multinucleated Langham's type giant cells surrounded by chronic lympho-plasmatic infiltration in the benign hepatocytes (Fig. 1, 2). Mycobacterial culture in L & J media did not produce any growth. Histopathological diagnosis was confirmed as tuberculous lesion by polymerase chain reaction (PCR) of biopsy sample. 

Further evaluation by sputum examination for AFB and Mantreux test, she had no evidence of acid-fast bacilli in the former and tuberculin test was negative. She was treated with CAT-1 anti-tubercular drug (ATD) therapy with extended continuation phase up to one year of therapy. She responded well in ATD and appetite improvement and weight gain was noted within two wk of therapy. She was followed up clinically as well as by USG and LFT at eight wk, 16 wk, six months and one year after starting therapy. She was completely asymptomatic after 8 wk of anti-tubercular therapy. The lesion was gradually reduced in size and vanished at six months. After one-year completion of ATD, she had no symptoms, no liver function abnormality and no evidence of tuberculous lesion. 

## Discussion

Isolated hepatic TB is the localized form of hepatic TB. Hepatobiliary TB is uncommon; but not exceptional (5, 6). The tuberculous lesions of liver are classified in three categories: secondary to miliary tuberculosis, granulomatous /tubercular hepatitis and localized hepatic TB (1, 2). Hepatic TB secondary to miliary TB accounts the largest number of cases (50-80%) of hepatic TB (1, 2). Isolated localized tuberculosis/localized tuberculoma are rarest type of hepatic TB accounting to 0.3% of all hepatic TB (4, 7). Most of the cases of localized hepatic TB in the world literature are 30-50 year age group and commonly present with abdominal pain (45-66%), fever (50-90%) and hepatomegaly (1, 2). When localized hepatic TB involves bile duct, obstructive jaundice will be the clinical presentation (1). *Mycobacterium* TB may reach to hepatobiliary system either by hematogenous route gastrointestinal foci, lymphatics or due to ruptured tuberculous lymph nodes near the portal tract. However, localized hepatic TB is either because of spread of tuberculous bacilli via lymphatics or due to rupture of tuberculous lymph nodes near portal tract (1, 2).

Diagnosis of localized hepatic TB is always difficult. Liver function tests often show abnormalities (elevated liver enzymes, hypoalbuminemia, hypogammaglobulinemia); however, these are non-specific and do not help in diagnosis (1, 2). In our cases also, liver function test did not help in specific diagnosis. Sometimes, chest X-ray of patients hepatobiliary TB may show old Koch’s lesions but active pulmonary TB may be found only in <10% cases (1). In our case, we could not find any radiographic and microbiological (sputum for acid-fast bacilli) evidence of pulmonary tuberculosis. A similar case of isolated was reported hepatic TB without any pulmonary Koch's lesion (4). In localized hepatic TB, ultrasound examination of liver shows nodular hypoechoic lesion or complex mass lesion simulating primary or metastatic tumor (8). 

CT scan has some fallacies like ultrasound simulating a tumor. In our case, both the ultrasound and CT scan misled us as a primary hepatic neoplasm or solitary metastatic nodule. Image-guided FNAC can confirm the diagnosis, but in our case yield was only necrotic material and acid-fast bacilli were not found in Ziehl Neelsen staining. Liver biopsy, either by image guidance or laparoscopic procedure and histopathology is very effective tool for diagnosis of localized hepatic tuberculosis. Histopathological features of localized hepatic TB show caseation necrosis, multiple will form epithelioid granulomas and presence of Langhans giant cells (1, 2). Caseation granulomatous lesion in liver biopsy is also seen in brucellosis, coccidioidomycosis, and Hodgkin’s disease but clinical presentation is different (2, 9). 

In the absence of acid-fast bacilli, we should not distract from the diagnosis of tuberculosis, especially in an endemic region (5). Diagnostic yield of laparoscopy was 88% and in laparoscopic biopsy, with histopathology, the yield was 100% (2). Mycobacterial culture and PCR assay of biopsy material have good diagnostic yield (5). In our case, mycobacterial culture was negative but PCR was positive for Mycobacterium tuberculosis. Treatment of hepatobiliary TB is similar to pulmonary tuberculosis. Initial four-drug regimen (HRZE) for two months and continuation phase (with two drugs, HR) up to one year is recommended to prevent drug resistance (1). Biliary decompression by stent placement during ERCP sometimes may be needed. 67% of cases respond well in 4 drug regime and have a well clinical response. In spite of response, overall mortality is 12%-42% and much higher in cases with jaundice (1). Adverse prognosis factors include younger age (<20 yr), acute presentation, coagulopathy, etc.

## Conclusion

Isolated hepatic tuberculosis, though a rare disease, can cause diagnostic difficulty due to similarity with nodular lesions of liver in imaging. Image-guided or laparoscopic biopsy and histopathology confirm the diagnosis. 

## Conflict of Interests

The authors declare that there is no Conflict of Interests. 
